# Human Tubal-Derived Mesenchymal Stromal Cells Associated with Low Level Laser Therapy Significantly Reduces Cigarette Smoke–Induced COPD in C57BL/6 mice

**DOI:** 10.1371/journal.pone.0136942

**Published:** 2015-08-31

**Authors:** Jean Pierre Schatzmann Peron, Auriléia Aparecida de Brito, Mayra Pelatti, Wesley Nogueira Brandão, Luana Beatriz Vitoretti, Flávia Regina Greiffo, Elaine Cristina da Silveira, Manuel Carneiro Oliveira-Junior, Mariangela Maluf, Lucila Evangelista, Silvio Halpern, Marcelo Gil Nisenbaum, Paulo Perin, Carlos Eduardo Czeresnia, Niels Olsen Saraiva Câmara, Flávio Aimbire, Rodolfo de Paula Vieira, Mayana Zatz, Ana Paula Ligeiro de Oliveira

**Affiliations:** 1 Neuroimmune Interactions Laboratory, Department of Immunology, Institute of Biomedical Sciences, University of Sao Paulo, São Paulo, SP, Brazil; 2 Laboratory of Pulmonary and Exercise Immunology–LABPEI, Nove de Julho University (UNINOVE), São Paulo, SP, Brazil; 3 Division of Human Genome Research Center, Biosciences Institute, University of São Paulo, São Paulo, SP, Brazil; 4 CEERH—Specialized Center for Human Reproduction, Division of Reproductive Medicine, São Paulo, SP, Brazil; 5 Division of Reproductive Medicine—Célula Mater, São Paulo, SP, Brazil; 6 Laboratory of Transplantation Immunobiology, Department of Immunology, Institute of Biomedical Sciences, University of São Paulo, São Paulo, SP, Brazil; 7 Department of Science and Technology, Federal University of São Paulo, São José dos Campos, SP, Brazil; University of Pittsburgh, UNITED STATES

## Abstract

Cigarette smoke-induced chronic obstructive pulmonary disease is a very debilitating disease, with a very high prevalence worldwide, which results in a expressive economic and social burden. Therefore, new therapeutic approaches to treat these patients are of unquestionable relevance. The use of mesenchymal stromal cells (MSCs) is an innovative and yet accessible approach for pulmonary acute and chronic diseases, mainly due to its important immunoregulatory, anti-fibrogenic, anti-apoptotic and pro-angiogenic. Besides, the use of adjuvant therapies, whose aim is to boost or synergize with their function should be tested. Low level laser (LLL) therapy is a relatively new and promising approach, with very low cost, no invasiveness and no side effects. Here, we aimed to study the effectiveness of human tube derived MSCs (htMSCs) cell therapy associated with a 30mW/3J—660 nm LLL irradiation in experimental cigarette smoke-induced chronic obstructive pulmonary disease. Thus, C57BL/6 mice were exposed to cigarette smoke for 75 days (twice a day) and all experiments were performed on day 76. Experimental groups receive htMSCS either intraperitoneally or intranasally and/or LLL irradiation either alone or in association. We show that co-therapy greatly reduces lung inflammation, lowering the cellular infiltrate and pro-inflammatory cytokine secretion (IL-1β, IL-6, TNF-α and KC), which were followed by decreased mucus production, collagen accumulation and tissue damage. These findings seemed to be secondary to the reduction of both NF-κB and NF-AT activation in lung tissues with a concomitant increase in IL-10. In summary, our data suggests that the concomitant use of MSCs + LLLT may be a promising therapeutic approach for lung inflammatory diseases as COPD.

## Introduction

According to the World Health Organization (WHO), more than five million deaths per year are the result of direct use of tobacco, whereas more than 600 000 second hand smokers also perish from cigarette exposure (World Health Organization, 2014). According to the US Centers for Disease Control and Prevention, cigarette smoking is the dominant risk factor for the development of chronic obstructive pulmonary disease (COPD) and emphysema [1,2]. A wide range of dangerous agents are found in cigarette smoke and, aside from solid particles, it includes more than 4000 chemicals, of which at least 250 are known to be highly harmful and more than 50 are tumorigenic [2]. Altogether, these factors may induce airway inflammation, cellular recruitment, lung fibrosis, mucus hypersecretion and also cancer [2,3]. Among the features of cigarette smoke-induced pathology, parenchymal fibrosis and emphysema may be considered the most characteristic and also the most deleterious, resulting in a significant organic impairment and restricted life quality of the patients [3,4].

Lung inflammation induced by cigarette smoke is characterized by an initial phase of inflammatory cells recruitment to the parenchymal space, matrix metalloproteinase (MMPs) activation, extracellular matrix degradation and tissue damage [3,4]. This is followed by the intense release of pro-inflammatory cytokines, such as IL-1 [5] and IL-6 [3], chemokines as CCL2 and CXCL1 and also lipid mediators, as PGI and LTB_4,_ as reviewed [2]. These molecules are able to recruit more inflammatory cells, as neutrophils, macrophages and also CD4 and CD8 T lymphocytes of the Th1 and Tc1 IFN-γ-secreting subtypes, respectively, perpetuating lung tissue damage [6]. Chronic exposure to cigarette noxious agents leads to the perpetuation of the inflammatory and fibrotic processes, with more and more infiltrating cells recruited, progressive elastin and collagen degradation, followed by parenchymal destruction and finally the establishment of lung emphysema [3,7]. Thus, campaigns to prevent cigarette smoking and to alert the population about its morbidity are extremely important. However the need for effective and also less expensive therapeutic approaches for the treatment of cigarette smoke-induced emphysematous patients is unquestionable. In this context, mesenchymal stromal cells (MSCs) therapy seem very promising, not only due to its regenerative capacity and immunomodulatory features, but also due to its low cost and relatively easy manipulation, as reviewed [4].

Adult MSCs are typically defined as undifferentiated multipotent cells endowed with the capacity for self-renewal and the potential to differentiate into several distinct cell lineages, as recently reviewed [8]. These cells may be obtained from different organs and tissues, as bone marrow [9,10], skeletal muscle [11], adipose tissue [12,13], dental pulp [14], umbilical cord [15,16], fallopian tubes [17] and other tissues. It is noteworthy that MSCs are well tolerated *in vivo*, either under syngeneic [18–19] or xenogenic conditions [20, 21, 22]. Concerning its immunomodulatory function, MSCs are known to: i) secrete anti-inflammatory cytokines; ii) express reduced levels of MHC and costimulatory molecules; iii) express the tryptophan-depleting enzyme indoleamine-2,3-dioxigenase [23] iv) induce T regulatory cells [18] and many others. Altogether, these mechanisms may be useful tools for the treatment of several chronic inflammatory diseases such as multiple sclerosis, arthritis, diabetes, lupus, and also COPD and emphysema [24–26]. Moreover, the possibility to use associated approaches able to boost the overall response must be investigated.

In this sense, Low Level Laser Therapy (LLLT) seem to be an interesting approach, specially due to its non-invasiveness, lack of secondary effects, low cost [27] and ability to promote stem-cell proliferation *in vitro* [28]. LLLT has already shown interesting results for different human diseases, as oral mucositis [29], coronary disease [30] and also in experimental models, as for muscle dystrophy [31], asthma [32] and articular inflammation in mice [33]. Actually, our group has recently demonstrated that LLLT is able to reduce lung inflammation both during the asthma model [32] as well as secondary to gut ischemia Among the possible mechanisms proposed, we have recently demonstrated that alveolar macrophages irradiated with LLL had augmented AMPc synthesis, and this is able to reduce NF-κB activation and thus IL-6 and IL-1 secretion [21]. In this context, we decided to evaluate the effectiveness of associating human tubal mesenchymal stromal cells (htMSCs) and LLLT using the murine model of cigarette smoke-induced COPD. It is worthy to mention that we have previously reported that htMSCs display stemness properties and also miogenic and adipogenic capacity to differentiate [17].

Here, our results demonstrate that the co-therapy with htMSCs and LLLT are very effective in lowering important pulmonary inflammatory parameters, as cytokine secretion, cellular infiltrate of leukocytes, mucus secretion, collagen deposition and the activation of important transcription factors, as NF-κB and NF-AT. Altogether, we show that the association of stem cells therapy with LLL irradiation are clinically beneficial, and thus, may be considered as further interesting therapeutic approach.

## Materials and Methods

### Cigarette Smoke–Induced Emphysema and Treatment

Female C57BL/6 mice (6–8 weeks) were exposed to cigarette smoke during 75 days by whole body exposure using an adapted protocol from Biselli et all [34]. All experiments were performed on day 76. Briefly, animals were exposed to 14 commercially available cigarettes (containing Alcatrão 13mg, nicotine 1,10 mg and carbon monoxide 10 mg each) divided on 7 cigarettes in the morning and 7 cigarettes in the afternoon and each session lasted for 30 minutes. LLL irradiation with diode laser (30mW/3J at 660 nm) was performed twice a day from day 60 to day 75 (one hour after each cigarette exposure) during 180 seconds on the skin over the right upper bronchus with a spot size of 0.785 cm^2^. htMSCs (1x10^6^ cells) were infused on days 60 and 67. Experimental groups were divided in 7 groups, as follow: 1) Basal 2) COPD 3) COPD + LLL 4) COPD + MSCs (i.p) 5) COPD + MSCs (i.n) 6) COPD + MSCs (i.p.) + LLL 7) COPD + MSCs (i.n.) + LLL. We declare that all experiments were approved by the UNINOVE animal research committee (Comitê de Ètica no uso de Animais—CEUA—#AN005/2013) and human research committee (Comitê de Ética em Pesquisa com Seres Humanos (CEPSH) of University of Sao Paulo (# CEP-IBUSP-106/2010).

### htMSCs Isolation and Culture

Human tubal tissue was obtained from women (age 35–53 years) submitted to hysterectomy or tubal ligation/resection surgery. Samples were collected during the proliferative phase from fertile women who had not undergone exogenous hormonal treatment within the last three months. htMSCs experiments were approved by both the ethics committee of the Biosciences Institute of the University of São Paulo and the UNINOVE–Universidade Nove de Julho. Each sample was collected in HEPES-buffered Dulbecco Modified Eagle Medium / Hams F-12 (DMEM/F-12; Invitrogen, Carlsbad, CA) with 100 IU/mL penicillin (Invitrogen) and 100 IU/mL streptomycin (Invitrogen, Carlsbad, CA), maintained at 4°C and processed within 24 hours period. All samples were washed twice in phosphate saline buffer (PBS, Gibco, Invitrogen, Carlsbad, CA), finely minced with a scalpel and transferred to a 50 mL tube, and dissolved with collagenase IV (Sigma-Aldrich) at 0,1% diluted in PBS (Invitrogen) for 15 minutes, at 37°C, in a water bath. Further, samples were washed with 10 mL of DMEM/F-12 and added with 15 ml of pure TripLE Express, (Invitrogen, Carlsbad, CA) for 15 minutes at 37°C in a water bath. Subsequently, TripLe was inactivated with DMEM/F-12 supplemented with 20% FBS, 100 IU/mL penicillin and 100 IU/mL streptomycin, and pelleted by centrifugation at 400g for five minutes at room temperature. Supernatant was removed with a sterile Pasteur pipette. Cells were then plated in DMEM/F-12 (5mL) supplemented with 20% FBS, 100 IU/mL penicillin and 100 IU/mL streptomycin in plastic flasks (25cm^2^), and maintained in incubator with controlled humidified atmosphere of 5% CO_2_ at 37°C. The medium used for expansion was initially changed every 72 hours and routinely replaced twice a week thereafter. Data concerning osteogenic, chondrogenic and adipogenic differentiation, as well as cellular phenotype were previously published by our group [17] demonstrating the stem cell potential of this population. Human tubal tissue was obtained after written and signed informed consent approved by Comitê de Ética em Pesquisa com Seres Humanos (CEPSH) of University of Sao Paulo (# CEP-IBUSP-106/2010) and maintained at the Division of Human Genome Research Center, Biosciences Institute, University of São Paulo, São Paulo–SP—Brazil.

### Bronchoalveolar Lavage Fluid Cells and Cytospin

After euthanasia, bronchoalveolar lavage fluid was obtained as previously described [35]. Briefly, 1.5 mLs of PBS as intra-tracheally injected in the lungs and 1 mL was re-collected and centrifuged at 450 g 4°C during 5 minutes. Supernatants were discarged and the pellet suspended in the desired amount of PBS 2% FBS. Total cell count was performed in Newbauer chambers and differential cell count after cytospin protocol. For cytospin, aliquots of 100 μL were centrifuged at 300 g for 5 minutes. Samples were stained by May-Grunwald-Giemsa method and 300 cells per sample were counted on a blind fashion.

### Lung Mononuclear Cells and Flow Cytometry

Lungs were obtained after right heart perfusion of 10 mLs of cold PBS. The tissue was minced with scissors and incubated for 45 minutes at 37°C with 2,5 mg/mL of Colagenase D (Roche) in HBSS. To stop collagenase, cells were suspended in Ca^2+^/Mg^2+^ free HBSS and centrifuged at 450 g and 4°C for 5 minutes. Cellular pellet was suspended in 5–6 mLs of 37% Percoll (GE) in HBSS and gently laid over 5–6mLs of 70% Percoll in HBSS using 15 mLs conical falcon tubes. Samples were centrifuged at 950 g and 4°C for 30 minutes without breaks. Cell-containing ring was collected from the percoll gradient interface and suspended in PBS 2% FBS and centrifuged again at 450 g and 4°C for 5 minutes. Further, cells were suspended in PBS 2% FBS, counted and used as desired. For flow cytometry analysis, 5x10^5^ cells were first incubated with 0,25 μg of anti-CD16/32 at 4°C for 20 minutes to avoid inespecific binding. Cells were then stained with 0,25 μg of anti-CD4 APC and 0,25 μg of anti-CD8 PE in 25 μL of PBS 2% FBS for 20 minutes at 4°C. In the next step, cells were washed twice with 200 μL of PBS 2% and finally suspended in paraformaldehyde 1%. Cells were acquired in the flow cytometer Accuri C6 (BD Biosciences). 5x10^3^ T CD4^+^ events were collected after elimination of cell doublets by FSH-height x FSC-area plot analysis.

### Cytokine Secretion

BAL lavage fluid was centrifuged at 450 g and 4°C during 5 minutes. Supernatants were used to evaluate the presence of IL-1β, IL-6, IL-10, TNF-α, IFN-γ and KC by the ELISA method according to manufacturer instruction (R&D System).

### Histomorphometric Analysis of Inflammation, Collagen and Mucus

After perfusion, lung samples were maintained in formaldehyde 4% for inclusion in paraffin. Slices were stained with Siriud Red for collagen detection or Periodic Acid Schiff (PAS) for the detection of mucus. Were analyzed 15 airways per each animal under a 400X magnification. H&E staining was used for the analysis of peribronchial infiltrate and mean linear intercept measurement [36]. Morphological features were analyzed using the Image Pro Plus 4.5 (Media Cybernetics, Rockville, MD, USA).

### Peribronchial Inflammation

The area between the airway basal membrane and the airway adventitia was quantified using the software Image Pro-Plus and the number of mononuclear and polymorphonuclear cells was quantified in this area according to the morphological criteria. The results were expressed as the number of mononuclear and polymorphonuclear cells per square millimeter.

### Mucus Production and Collagen Fibers Deposition

For the analysis of mucus production, the epithelium area of the whole airway, 15 airways per mouse were quantified. The positive stained area in the epithelium area was quantified and the results were expressed as the percentage of positive epithelial area. For the analysis of collagen fibers deposition in the airways wall, the area between the outlayer of epithelium until airway adventitia were measured and the amount of positive stained area was calculated. The results were expressed as percentage aof collagen fibers in the airway wall [37–39].

### Immunohistochemistry

For immunohistochemistry analysis, all samples were previously submitted to deparaffinization. Endogenous peroxidase activity was blocked with H_2_O_2_ 3% three times during 10 minutes. Samples were then washed and blocked with bovine serum albumin 10% during 1 hour and then incubated with primary rabbit anti-mouse IgG antibodies: i) anti-NFκB (Santa Cruz, CA) at 1:500; ii) anti-NF-AT 1:500 (Santa Cruz, CA) and iii) anti-IL-10 (Santa Cruz, CA) at 1:500 during 2 hours at room temperature. Samples were washed twice with TBS- BSA 10% and then incubated with secondary antibody goat anti-Rabbit IgG at 1:1000 for 1 hour. After washing samples twice with BSA 2% diaminobenzidine (DAB) was added. Finally samples were washed again and counter stained with H&E. Slides were analyzed under light microscope on a blind fashion.

### Statistical Analysis

All analysis were performed using the GraphPad Prism software (GraphPad Software, Inc). For parametric analysis we used one-way ANOVA followed by Tukey post-test whereas for non-parametric analysis, we used Kruskal-Wallis followed by Dunn´s post-test. All groups were compared to COPD control group. Differences were considered significant when p<0,05.

## Results

### htMSCs and LLLT Reduce Bronchoalveolar Lavage Fluid Cellularity in the Lungs of COPD Animals

**Fig 1:**
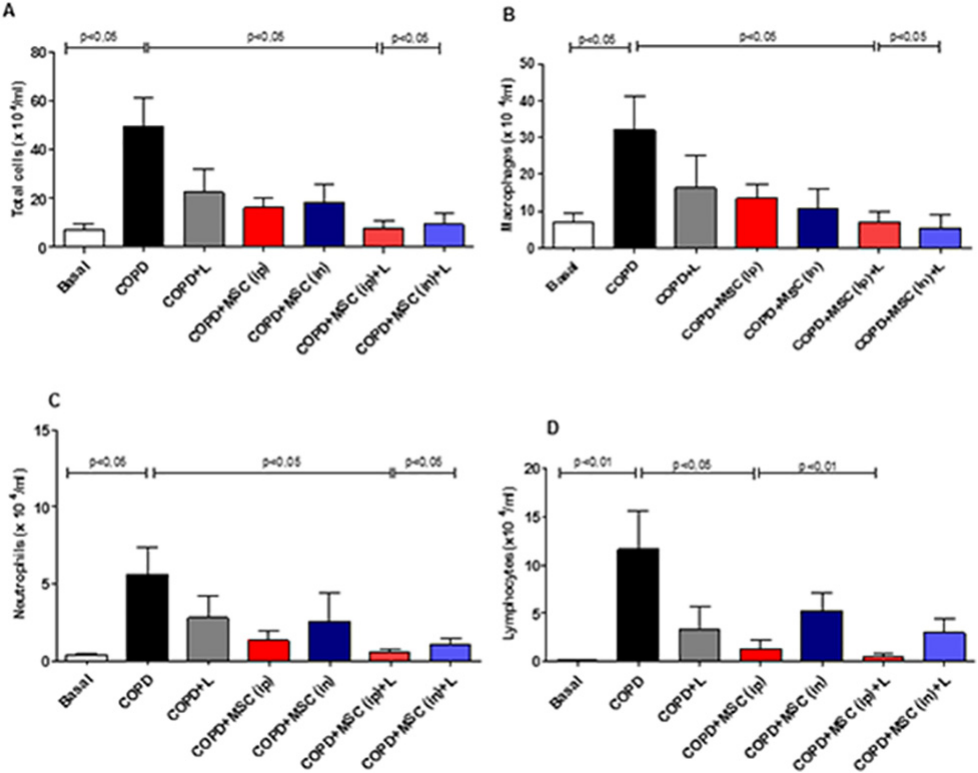
Reduced cellular infiltration in the lungs of htMSC and LLLT treated COPD animals. COPD animals were submitted to therapeutic protocols as described in materials and methods. Further, all animals were euthanized and BAL obtained for cytospin and the quantification of total cells (A), macrophages (B), neutrophils (C) and lymphocytes (D). Data representative of three independent experiments. n = 5–8 animals per group. One-way ANOVA.

To evaluate whether htMSCs and/or LLLT were effective in reducing lung inflammation after cigarette smoke-induced COPD, we first analyzed BAL fluid cellularity from all experimental groups for total and differential cell counts. Interestingly, a significant reduction in total cell counts, macrophages and neutrophils was observed only when htMSCs were associated with LLL therapy, although a trend is observed in all other groups, even for LLL alone (Fig 1A, 1B and 1C). However, we had a discrepant result for lymphocyte counts, as we observed that only htMSCs by i.p. delivery was able to reduce its amount. Association with LLLT did not changed the result. It is worthy to mention that the lowest amount of total cells (Fig 1A), macrophages (Fig 1B) and neutrophils (Fig 1C) was observed in the group of htMSCs associated with LLLT, either i.p or i.n, demonstrating that association with LLLT is more important for these features than the route of htMSCs delivery. It is also noteworthy that such reduction in BAL cellularity reached levels no different from naïve controls. Moreover, intraperitoneal delivery of htMSCs seemed more effective than intranasal delivery in reducing neutrophil and lymphocyte infiltration.

**Fig 2:**
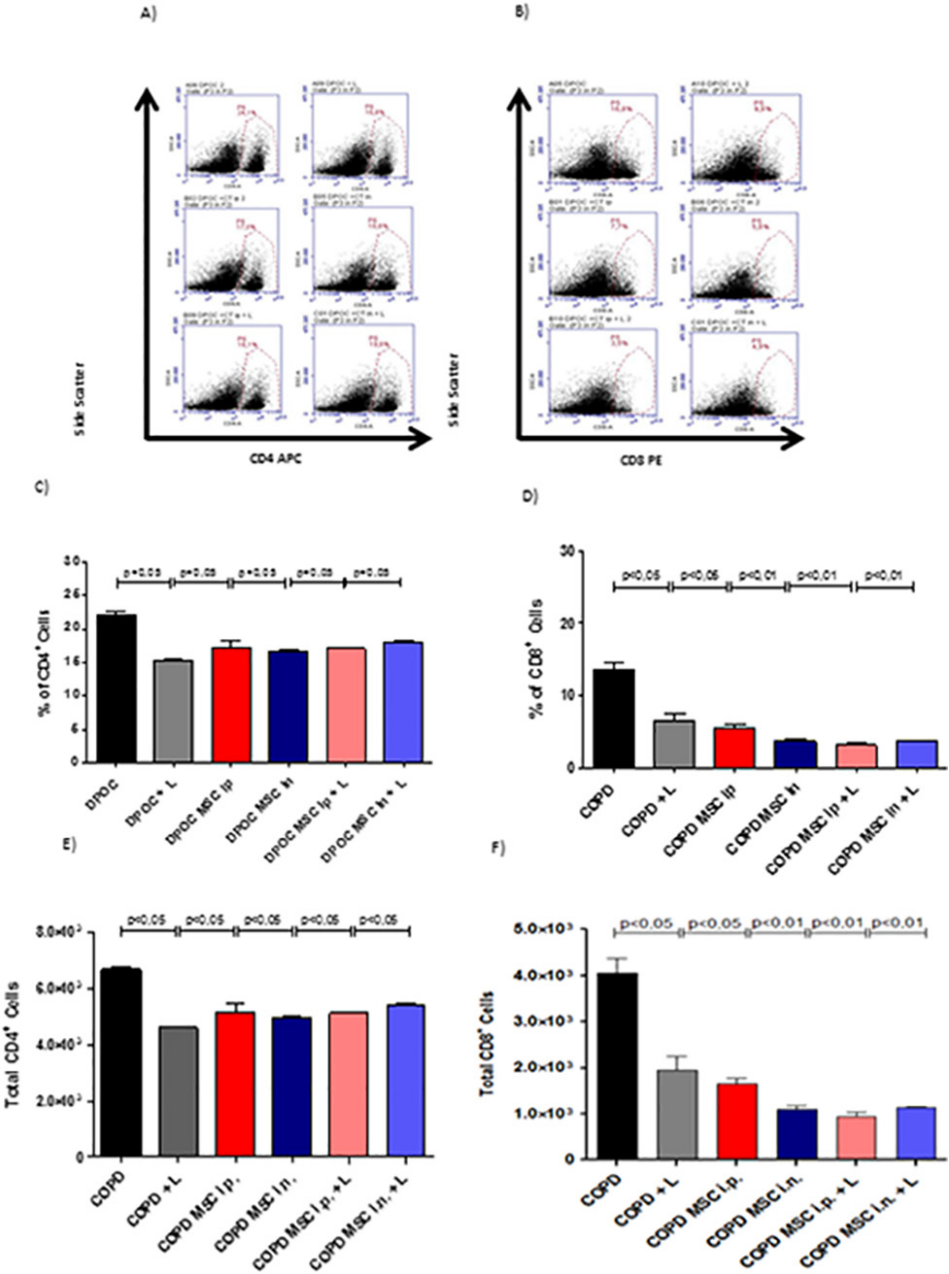
Reduction of T CD4^+^ and T CD8^+^ T cells infiltration in the lungs of htMSC and/or LLLT treated animals. COPD animals were submitted to therapeutic protocols as described in materials and methods. Further, all animals were euthanized and BAL obtained for flow cytometric analysis of the frequency of CD4^+^ and CD8^+^ T cells. In (A and B), dot plots depict the gates used. In (C and D), graphs of the percentage of CD4^+^ and CD8^+^ T cells, respectively. In (E and F),graphs of the absolute numbers of CD4^+^ and CD8^+^ T cells, respectively. Data representative of two independent experiments. n = 5–8 animals per group. One-way ANOVA.

Next, we performed flow cytometry analysis in the BAL fluid from all experimental groups to evaluate changes in the frequency of CD4^+^ and CD8^+^ T cells in the lungs. This could be indicative, either of a reduced T cell activation in the lymph nodes, or an impairment in cell migration to the target organ. In fact, corroborating findings from Fig 1 for polymorphonuclear and total cells, the frequency of both T CD4^+^ (Fig 2A and 2B) and T CD8^+^ (Fig 2C and 2D) cells were significantly reduced irrespective of the therapeutic approach used, i.e, htMSCs alone or in association with LLLT. However, no differences were observed, when lung tissue was analyzed for perivascular infiltrate by immunohistochemistry (S1 Fig).

### htMSCs and/or LLLT Reduce Inflammatory Cytokines in the BAL Fluid of COPD Animals

**Fig 3:**
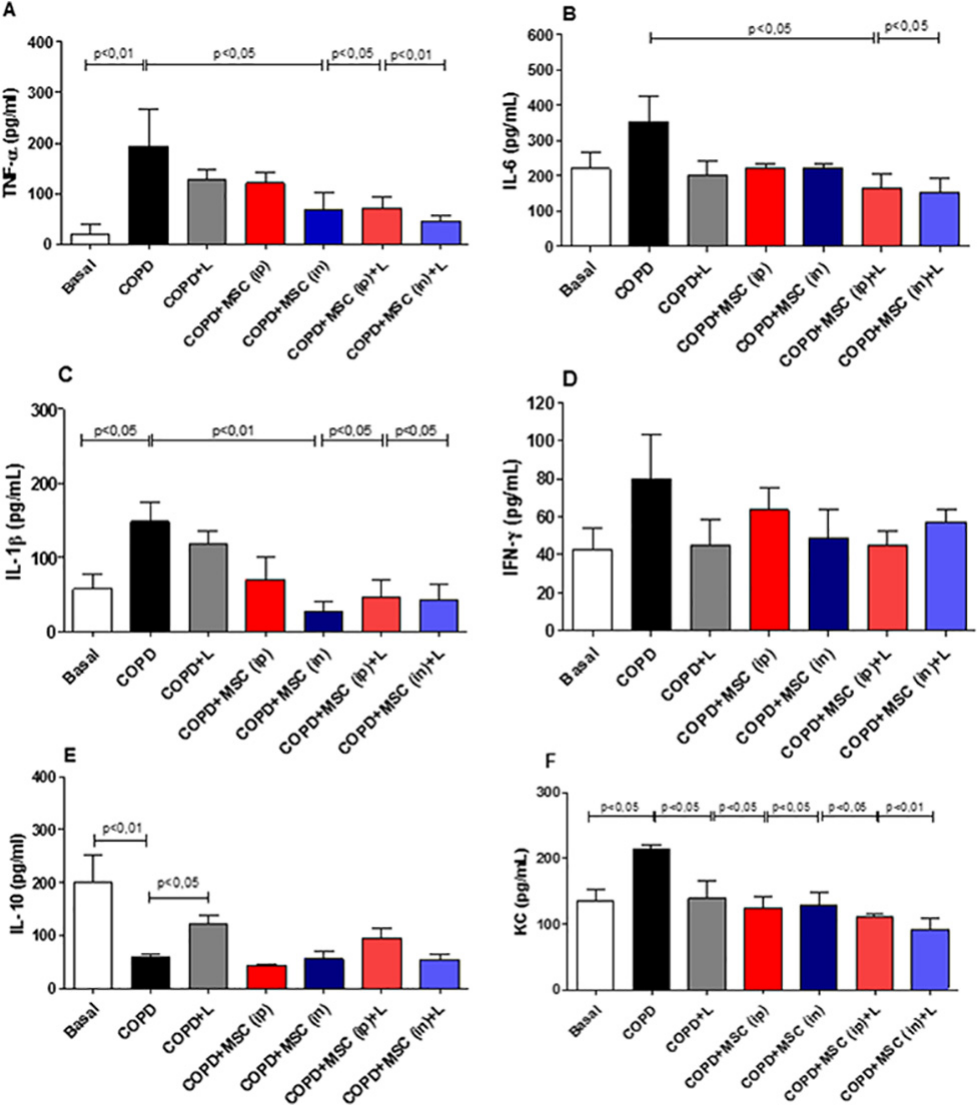
Reduced cytokine secretion in the lungs of htMSC and/or LLLT treated animals. COPD animals were submitted to therapeutic protocols as described in materials and methods. Further, all animals were euthanized and BAL obtained for cytokine detection by the ELISA method for A)TNF-α, B)IL-6 C)IL-1β D)IFN-γ E) IL-10 and F)KC. Data representative of three independent experiments. n = 5–8 animals per group. One-way ANOVA.

We next analyzed the amount of cytokines as IL-1β, IL-6, TNF-α, IFN-γ and the chemockine KC, to evaluate whether reduced cellularity in the lungs of our experimental culminated also in reduction of inflammatory mediators. Our results show that IL-1β was reduced when htMSCs either ip. or i.n. were associated with LLLT, although htMSCs i.n alone also reached significance (Fig 3C). The same pattern was observed for TNF-α (Fig 3A). KC, an important chemokine for the recruitment of neurotrophils, was reduced in all groups irrespective of the treatment (Fig 3F). Interestingly, IL-6 was reduced only when htMSCs were associated with LLL (Fig 3B) whereas no difference was observed for IFN-γ (Fig 3D) Very interestingly, the anti-inflammatory cytokine IL-10 was up-regulated only with LLL irradiation alone (Fig 3E).

### htMSCs and/or LLLT Reduced Mucus Secretion and Alveolar Enlargement in the Lungs of COPD Animals

**Fig 4:**
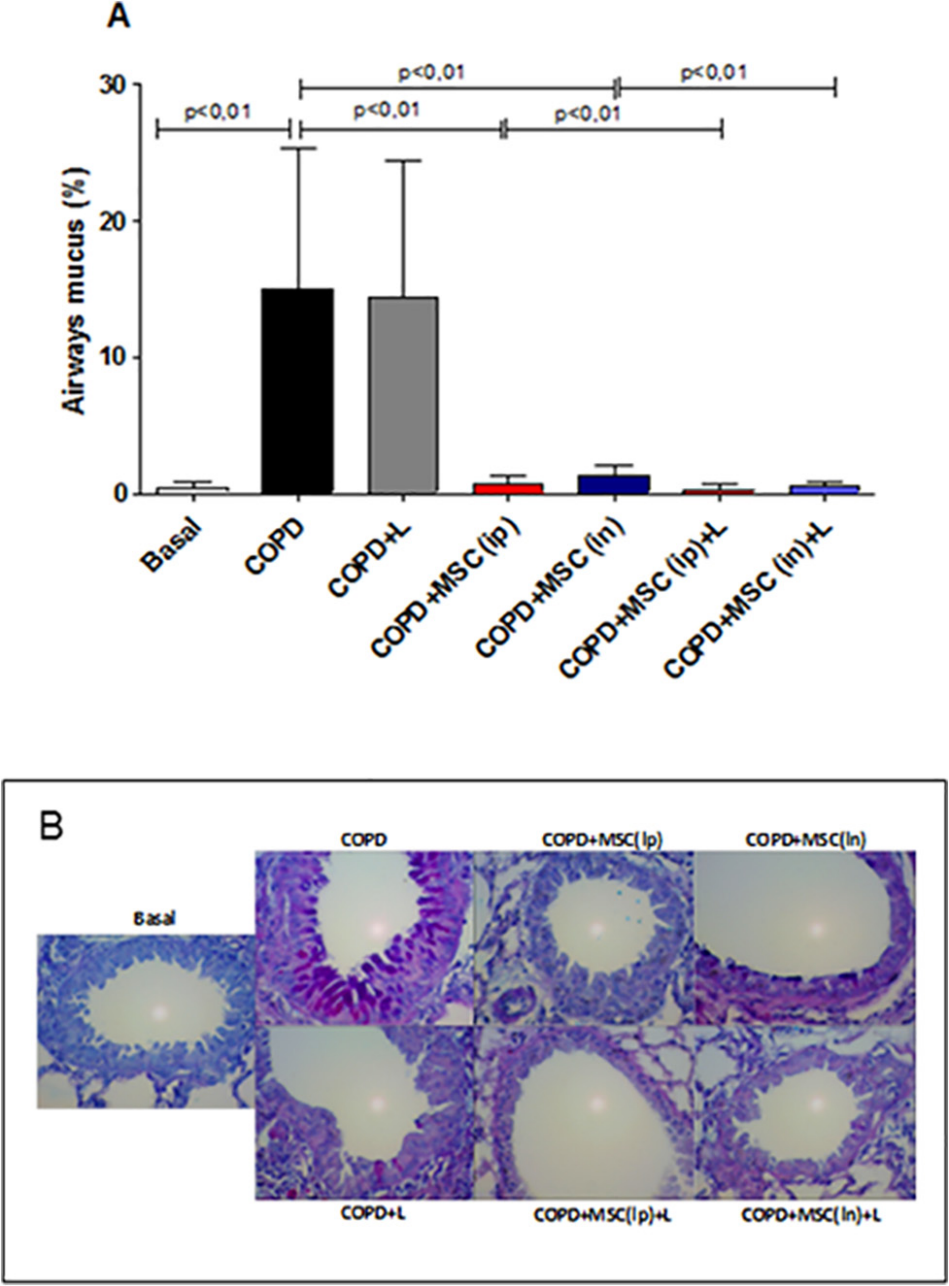
Reduced mucus secretion in the lungs of htMSC and/or LLLT treated animals. COPD animals were submitted to therapeutic protocols as described in materials and methods. Further, all animals were euthanized, lungs were obtained and sections were stained by PAS (Periodic Acid Schiff) as described in methods. In A) representative graphs and B) photomicrographs of PAS stained sections. Data representative of two experiments. n = 5–8 animals per group. One-way ANOVA.

Another important feature in the pathophysiology of COPD is the intense secretion of mucus, resulting in reduced airway lumen, airflow and breathlessness. Thus, we next evaluated whether either htMSCs or LLL therapies, associated or not, were able to reduce the amount of mucus observed in the airways. In fact, our data demonstrated significantly decreased amount of mucus in the bronchi of all experimental animals under therapeutic protocols, with the exception of the LLLT alone. Significant differences reached from 10 to 15-fold reduction when experimental groups were compared to COPD control. On the other hand, when associated with htMSCs, either nasally or intraperitoneally, LLL therapy restored mucus secretion to basal levels, with no difference from naïve animals (Fig 4A and 4B). We next decided to verify some possible effects of htMSCs and LLLT in the enlargement of lung parenchyma, which reflects the destruction of the alveolar septa. As indicated by S2 Fig, mean linear intercept measures were significantly reduced in the COPD + MSC(ip)+LLLT group when compared to control animals, indicating reduced lung damage.

### Collagen Deposition Was Reduced Only When htMSCs Were Associated with LLL

**Fig 5:**
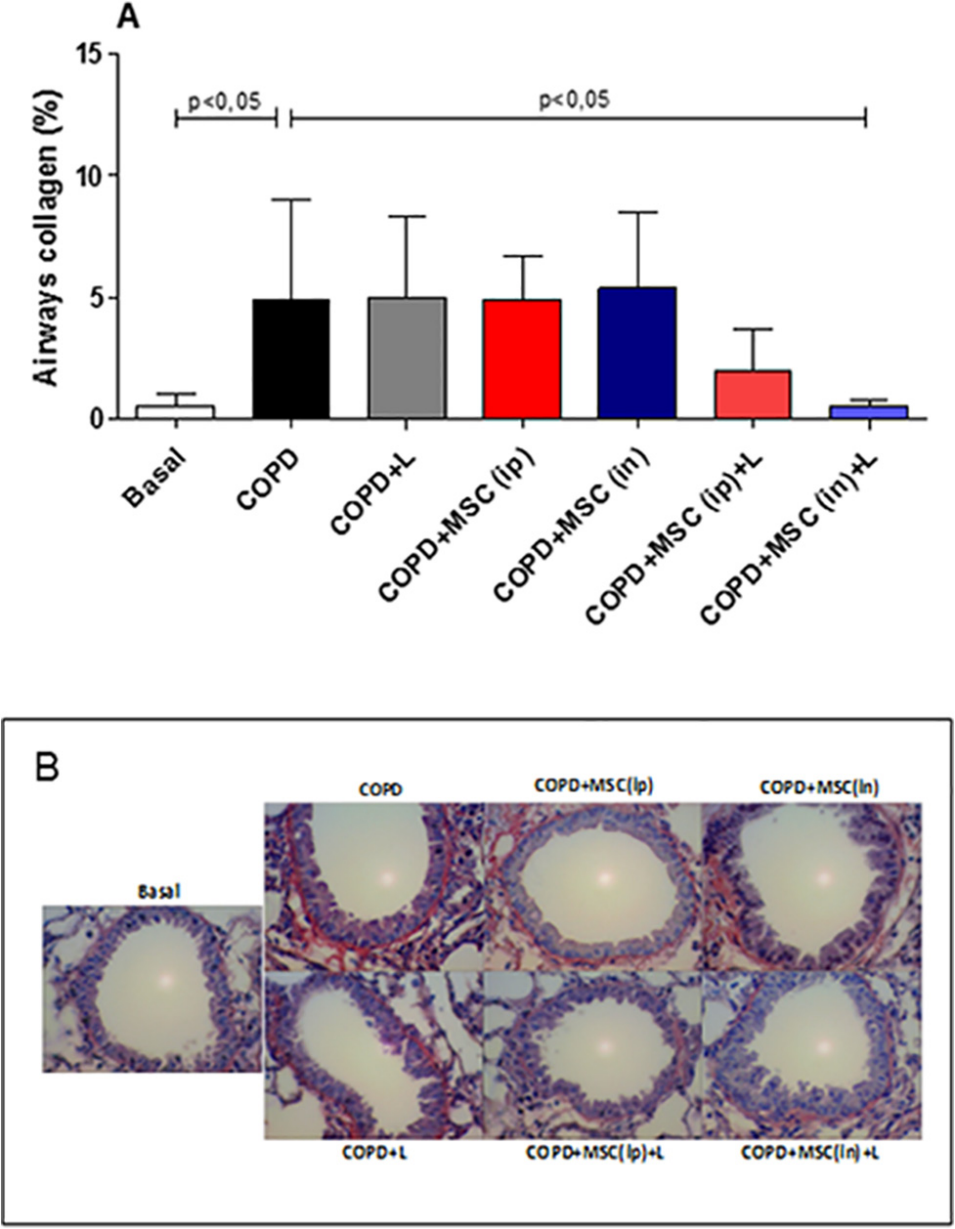
Reduced collagen deposition only in the lungs of htMSC i.n and LLL treated animals. COPD animals were submitted to therapeutic protocols as described in materials and methods. Further, all animals were euthanized and lungs were obtained and sections were stained with Sirus Red for collagen detection. In A) representative graphs and B) photomicrographs of Sirius Red stained sections. Data representative of two experiments. n = 5–8 animals per group. One-way ANOVA.

Aside from mucus secretion, collagen deposition is also considered an important marker for COPD. Therefore, we also evaluated the amount of peribronchial collagen deposition through Sirius Red methodology (Fig 5A and 5B). Very surprisingly, a significant collagen decrease of around 4-fold was observed only when LLLT was associated with htMSCs intranasally. Although there was a trend for the htMSCs (i.p) + LLLT, none of the other groups reached statistical significance (Fig 5B).

### htMSCs and/or LLLT Reduces NF-κB and NF-AT Transcription Factors Expression in the Lungs of COPD Animals

**Fig 6:**
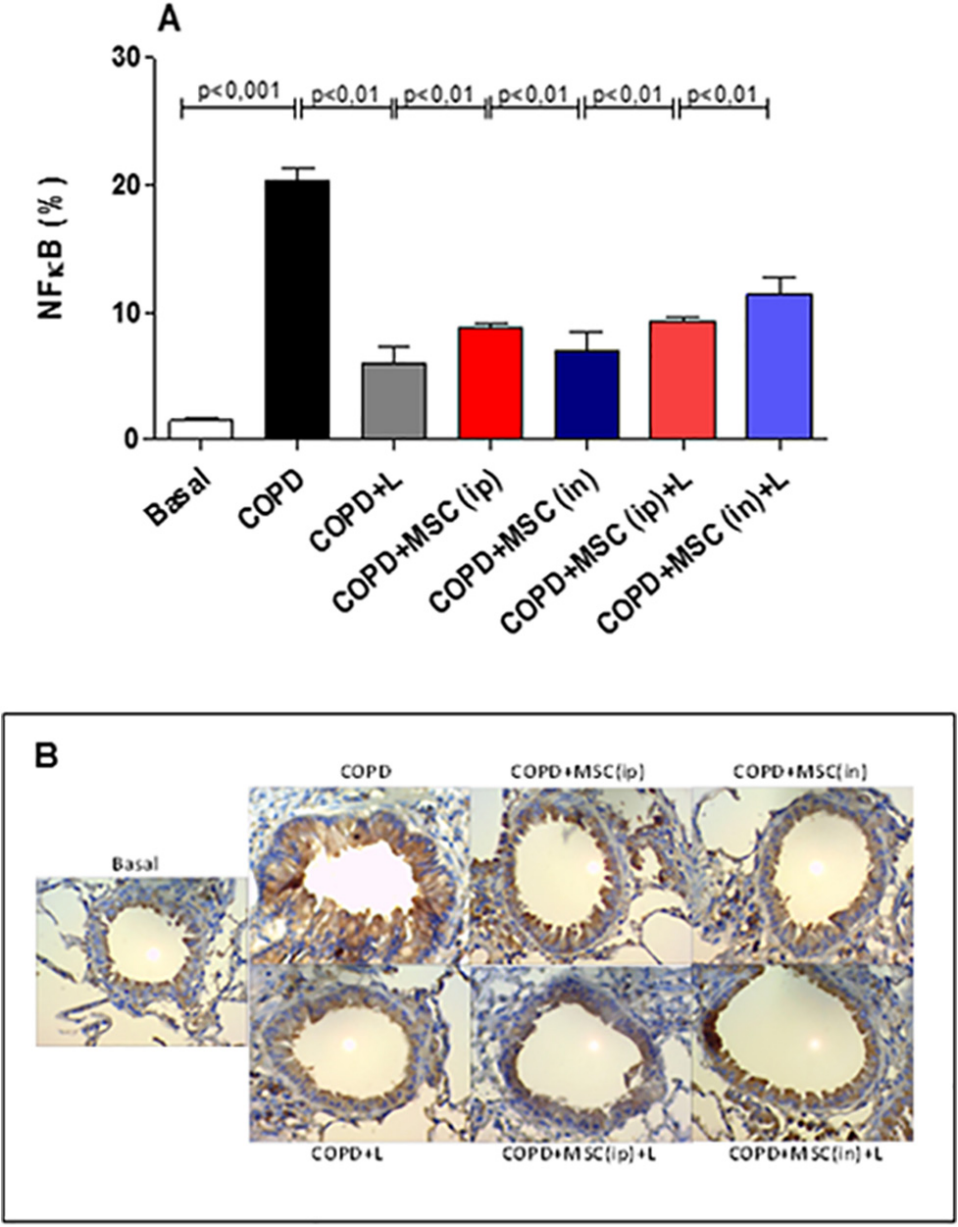
Reduced total NF-B staining in the lungs of htMSC i.n and/or LLLT treated animals. COPD animals were submitted to therapeutic protocols as described in materials and methods. Further, all animals were euthanized, lungs obtained and sections were stained with anti-NF-B In A) representative graphs and B) photomicrographs of immunohistochemistry stained sections. Data representative of two experiments. n = 5–8 animals per group. One-way ANOVA.

Most pro-inflammatory cytokines evaluated, such as IL-1, IL-6 and TNF-α, have their transcription under the control of the transcription factor NF-κB. Moreover, these cytokines may also signal through NF-κB after engagement with its receptors cognate recptors. NF-κB is found in a great variety of cell types, as macrophages and neutrophils, but also T lymphocytes. Thus, with the aim to correlate the overall reduction in BAL cellularity and pro-inflammatory cytokine secretion with a reduction in NF-κB expression, we sought to evaluate its expression by immunohistochemistry of the perialveolar space of COPD animals (Fig 6). Corroborating the reduction in pro-inflammatory cytokine secretion (Fig 3C), all groups displayed significantly reduced NF-κB staining in the target tissue when compared to the COPD control group (Fig 6B). Differences vary from 2 to 3-fold depending on the treatment.

**Fig 7:**
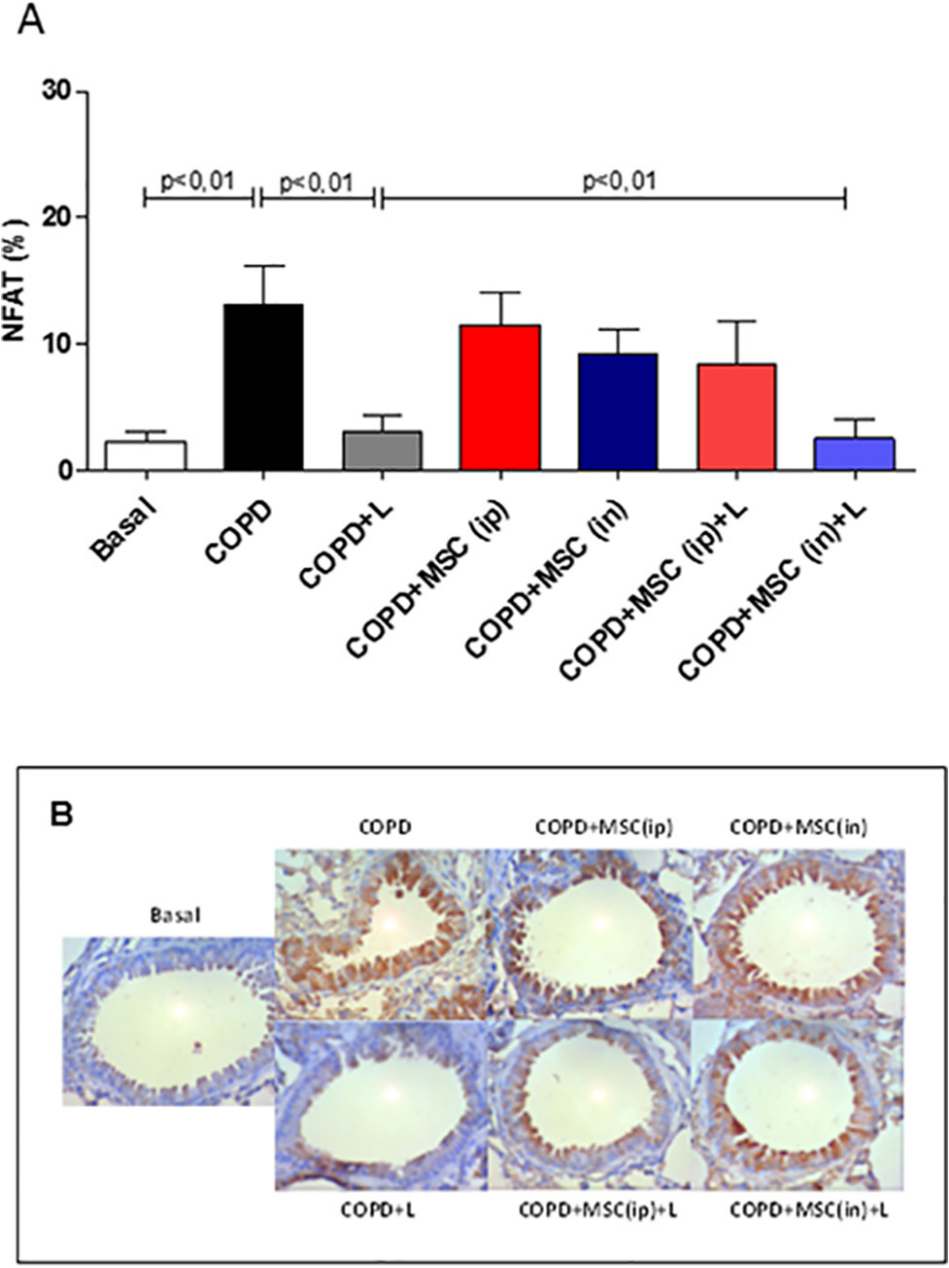
Reduced total NF-AT staining in the lungs of htMSC i.n and/or LLLT treated animals. COPD animals were submitted to therapeutic protocols as described in materials and methods. Further, all animals were euthanized, lungs obtained and sections were stained with anti-NF-AT. In A) representative graphs and B) photomicrographs of immunohistochemistry stained sections. Data representative of two experiments. n = 5–8 animals per group. One-way ANOVA.

Another transcription factor also important for the activation of the immune cells is the NF-AT, which is mostly expressed on T cells, both CD4^+^, CD8^+^ and NK cells, and also in the lung tissue, as shown by the literature [40]. NF-AT is the main transcription factor involved in the synthesis of IL-2 and its active form is the non-phosphorilated state, as reviewed [41]. It was very surprising to notice that LLLT by itself was already able to significantly reduce NF-AT activation (Fig 7A). This reduction was no different from that observed when LLL was associated with htMSCs i.n. (Fig 7A).

### LLL Therapy Boosts IL-10 Secretion in Lung Tissue

In order to confirm the data obtained by the ELISA method, and concerning its importance as an anti-inflammatory factor, we also evaluated by immunohistochemistry the amount of IL-10 in lung tissue of the experimental groups. Interestingly, LLL irradiation *per se* was able to increase the amount of epithelial IL-10 (S3 Fig) whereas htMSCs i.p. was not able to so. Consistently, this was also observed only when htMSCs were associated with LLL therapy, although htMSCs i.n. alone could also up-regulate this cytokine (S3 Fig).

## Discussion

In the present research we show that the treatment with htMSCs associated with LLL irradiation significantly reduced lung inflammation in mice with cigarette smoke-induced COPD. Many of the evaluated parameters, such as BAL cellularity, pro-inflammatory cytokine secretion, perivascular infiltrate and the presence of inflammatory transcription factors, as NF-κB and NF-AT were significantly reduced. This was associated with a better maintenance of tissue integrity when compared to COPD untreated controls, evidenced by reduced mucus secretion, collagen deposition and tissue damage. It is worthy to mention that these features are greatly responsible for tecidual destruction and further decline in patient´s life quality, as it greatly reduces airflow, lung complacence and lowering pO_2_ [2,42]. Moreover, our findings also demonstrate that the route of administration of htMSCs, i.e. intraperitoneal vs. intranasal, were both effective in suppressing the disease, although with some peculiarities. For instance, intranasal delivery was more effective in reducing the presence of NF-AT as well as collagen deposition when associated with LLL therapy, which was not observed after intra-peritoneal injection. This might be explained by a local regenerative/suppressive mechanism, as the cells were directly delivered to the lungs. For intra-peritoneal route, however, immune modulation on lymphoid organs, specially mediastinal lymph nodes is more likely.

Cigarette smoking-associated diseases, which may result in severe decrease of life quality represent an important social and economical burden, as billions of dollars are spent each year in the treatment of emphysematous patients worldwide. Despite the constant campaigns against tobacco, it is still the fourth leading cause of death in the United States [43]. Therefore, the need for a more efficient and yet cheaper therapeutic approach in the management of COPD patients is unquestionable. Cell therapy with MSCs has shown clinical benefits by us and other groups in conditions such as experimental autoimmune encephalomyelitis (EAE)[22], multiple sclerosis [44], chronic renal failure [13], ischemic cerebral stroke [9], myocardial stress [45] and even for COPD. In fact, several of our findings were corroborated by previous research, in which lung inflammation, weight loss and lung integrity were ameliorated after adipose-tissue MSCs treatment [26]. On the other hand, a recent clinical trial on MSCs have not shown promising results, as COPD patients treated with MSCs (Prochymal) had not shown improvement of lung function, as shown for forced expiratory volume (FEV_1_) and forced volume capacity (FVC). Interestingly however, it seemed that, corroborating our findings, lung inflammation was decreased, inferred by the lower level of C-reactive protein [46]. Our report however, although using an experimental model, is the first to associate htMSCs with LLL irradiation, considered a promising approach [27].

COPD is a chronic and obstructive disease of the lungs, result of the chronic exposure of the airways to the noxious agents found in cigarette smoke and droplets. It has been established that lung destruction directly correlates with the amount of total particulate matter found in each cigar [7]. Initial exposure leads to cellular infiltrate of neutrophils and blood-derived monocytes secreting pro-inflammatory and pro-fibrotic cytokines, as IL-1, IL-6, IL-12, TNF-α and TGF-β, along with chemokines, lipid mediators and several other molecules [2,3]. This is greatly accompanied by extracellular matrix degradation by MMP-1 (matrixmetalloproteinase-1) secreted by alveolar macrophages causing tissue destruction and emphysema [3]. Chronic exposure leads to intense T CD4 and T CD8 lymphocyte infiltration, and IFN-γ is probably the most important T cell-derived cytokine. It is relevant that IFN-γ-secreting T CD8 cells are also very important in the late phase of the disease [47]. Due to its pro-inflammatory function, and in association with other cytokines, as TNF-α, TGF-β, IFN-γ induces important activation of both immune and parenchymal cells of the lung and finally leading to fibrosis and parenchymal destruction [2,3]. In this context, therapeutic approaches with the capacity to dampen such activation could greatly contribute to the maintenance of lung integrity and both MSCs and LLL therapy independently had already proven it [10,32]. However, we did not observe differences for IFN-γ.

A growing body of evidence have shown several different immunosuppressive mechanisms of MSCS, such as: lowering the expression of MHC and costimulatory molecules; up-regulation of indoleamine-2,3-dioxigenase [19,48] and FAS-L expression [49]; expansion of Tregs [48]; block of IFN-γ and IL-17 secretion [22], reduces tecidual caspase-3 activation [10] and many others, as reviewed [4]. Altogether, these mechanisms greatly impair immune cells activation, further avoiding or reducing tissue inflammation and destruction. However, although we may only speculate the mechanisms used by the htMSCs + LLL irradiation to suppress COPD, it is suitable to think that many of the aforementioned mechanisms may be taking place in our system.

Corroborating our findings, several other groups have already addressed the capacity of MSCs in modulating chronic diseases, including COPD. Bone marrow-derived MSCs were shown to significantly reduce tissue destruction in COPD mice by a mechanisms dependent on VEGF. Consistently, the group observed among many other cytokines, reduction in IL-1β and IL-6, in accordance with our data. More relevant was the fact that lung function, including inspiratory capacity and vital capacity were significantly higher when compared to control animals, which is again discrepant from data obtained in humans [10].

Adipose tissue-derived MSCs (ASCs) transplantation was also shown to be effective in improving COPD in mice reducing lung infiltration of inflammatory cells, as neutrophils and macrophages reduced associated with parenchymal destruction, as evaluated by active caspase-3 [12]. In line with our findings, the group also demonstrated reduced lung infiltration of inflammatory cells, as total cells, neutrophils and macrophages.

Associated with its immunosuppressive activity, it is also possible that MSCs exert some regenerative/reparative function *in situ*. For instance, intra-tracheal delivery of bone marrow-derived stem cells after hyperventilation-induced injury in rats greatly improved lung recovery, reducing cellular infiltrate of neutrophils and lymphocytes, pro-inflammatory cytokine secretion, associated with normal alveolar space [50]. In accordance, we have observed that intra-nasally treated animals have also displayed a significant reduction in several inflammatory markers, as cellular infiltrate and cytokine secretion. Moreover, we also observed amelioration in alveoli enlargement in animals treated with htMSCs(ip) + LLL, evidenced by mean linear intercept, but unfortunately lung function was not assessed by us. However, it is noteworthy that collagen deposition, a very significant factor for tissue fibrosis and lung emphysema, was significantly reduced when htMSCs were associated with LLLT. This may indicate that LLLT boosts htMSCs regerative capacity, however we may not describe the mechanisms so far.

LLLT started to be studied in the late 60`s, when reports had shown its use in improving hair growing in rats. Several other reports soon followed using different models, as wound healing and muscle regeneration. On the other hand, the effect of laser therapy is poorly understood. We have previously observed that the *in vitro* proliferative capacity of dental pulp derived mesenchymal stem-cells increased with low intensity laser application [28]. Interestingly, recent reports have shown that LLL therapy exert its function by increasing intracellular AMPc and thus suppressing important inflammatory transcription factors as NF-κB [51], which is consistent with our findings. In fact, the intriguing capacity of LLL therapy in suppressing the immune response has been previously shown by us and others, as mentioned. LLLT irradiation had the capacity to reduce joint inflammation in rats treated locally with papain. Consistently, irradiated groups demonstrated reduced cellular infiltration and also reduction in IL-1β and IL-6, corroborating our findings [33]. In the context of lung disease, we have previously published that LLLT reduces lung inflammation using OVA-induced lung inflammatory disease [32]. In this case, several parameters, as cellular infiltrate of the lungs, cytokine secretion, mucus secretion and collagen deposition were significantly reduced after irradiation and antigen challenge. As expected, many of these results were reproduced in the present research, and also it indicated that htMSCs and LLLT may act by an additive manner.

Aside from overall reduction in lung inflammation, another interesting observation was the reduction of the transcription factors NF-κB and NF-AT which could be secondary to the reduced infiltration of macrophages / neutrophils and lymphocytes in lung tissue, respectively. Moreover, we have also detected lower levels of IL-1β in BAL fluid, which is known to signal through IL-1r and thus induce NF-κB activation. However, we may not exclude the possibility that LLLT could also directly abrogate NF-AT activation, as observed when it was used alone. It is plausible to think that LLLT somehow changes intracellular Ca^2+^ levels, consequently modulating NF-AT activation by calcineurin / calmodulin. It is relevant that NF-AT, which may also be found in lung arteries, is responsible for up-regulating the expression of smooth muscle α-actin and myosin heavy chain after hypoxia, a very important mechanism for chronic hypoxia-induced pulmonary vascular remodeling [52].

In summary, our results clearly indicate that, aside from lack of toxicity and other complications, both htMSCs and LLLT were shown to be safe for the treatment of COPD in an experimental model. More important, however, is the fact that although htMSCs and LLLT could act independently, some pathological parameters were effectively reduced when both therapies were associated. It may indicate an important additive effect that may be responsible for the overall reduction in lung inflammation and tissue destruction in cigarette smokers. Our results of the combined use of htMSCs and LLLT were effective in reducing inflammatory immune response and further overall destruction of lung parenchyma in experimental COPD. Thus, we encourage other groups to keep focus on the potential of MSCs in COPD, and also to consider the relevance of associating it with LLLT. This is not only to reinforce our data, but mainly to refine the therapeutic scheme with the aim to reach a more translational approach.

## Conclusions

Our research highlights the important suppressive capacity of htMSCs in reducing overall lung inflammation during COPD in mice. Besides, we also observed a beneficial additive effect when htMSCs and LLLT are associated. Reduced lung cellularity and cytokine secretion, mucus production, collagen deposition and transcription factors activation are among the downregulated parameters. Interestingly, our data also show that this phenomenom is very consistent, irrespective of the MSCs administration via, i.e. intraperitoneal or intranasal. In summary, our data highlights the possibility of using this approach for the treatment of chronic inflammatory lung diseases.

## Supporting Information

S1 Fig Analysis of polymorphonuclear and mononuclear perivascular cells in the lungs of htMSC and/or LLLT treated animals. COPD animals were submitted to therapeutic protocols as described in materials and methods. Further, all animals were euthanized and lungs were obtained for histomorphometric analysis. In (A) mononuclear and (B) polymorphonuclear cells per mm^2^ of tissue. Data representative from two experiments. n = 5–8 animals per group. One-way ANOVA.
(TIF)

S2 Fig Reduction in alveolar destruction in the lungs of htMSC and/or LLLT treated animals. COPD animals were submitted to therapeutic protocols as described in materials and methods. Further, all animals were euthanized and lungs were obtained for mean linear intercept in central and peripheral measures of both lungs. In A) representative graphs and B) photomicrographs of haematoxylin- and eosin-stained pulmonary parenchyma. Data representative of two experiments. n = 5–8 animals per group. One-way ANOVA.
(TIF)

S3 Fig Increased IL-10 staining in the lungs of htMSC i.n and/or LLLT treated animals. COPD animals were submitted to therapeutic protocols as described in materials and methods. Further, all animals were euthanized, lungs obtained and sections were stained with anti-IL-10. In A) representative graphs and B) photomicrographs of immunohistochemistry stained sections. Data representative of two experiments. n = 5–8 animals per group. One-way ANOVA.
(TIF)
