# Correction: Human Tubal-Derived Mesenchymal Stromal Cells Associated with Low Level Laser Therapy Significantly Reduces Cigarette Smoke-Induced COPD in C57BL/6 mice

**DOI:** 10.1371/journal.pone.0139294

**Published:** 2015-09-25

**Authors:** Jean Pierre Schatzmann Peron, Auriléia Aparecida de Brito, Mayra Pelatti, Wesley Nogueira Brandão, Luana Beatriz Vitoretti, Flávia Regina Greiffo, Elaine Cristina da Silveira, Manuel Carneiro Oliveira-Junior, Mariangela Maluf, Lucila Evangelista, Silvio Halpern, Marcelo Gil Nisenbaum, Paulo Perin, Carlos Eduardo Czeresnia, Niels Olsen Saraiva Câmara, Flávio Aimbire, Rodolfo de Paula Vieira, Mayana Zatz, Ana Paula Ligeiro de Oliveira

The affiliation for the eighth author is incorrect. Manuel Carneiro Oliveira-Junior is not affiliated with #3 but with #2 Laboratory of Pulmonary and Exercise Immunology–LABPEI, Nove de Julho University (UNINOVE), São Paulo, SP, Brazil.

The affiliation for the seventeenth author is incorrect. Rodolfo de Paula Vieira is not affiliated with #3 but with #2 Laboratory of Pulmonary and Exercise Immunology–LABPEI, Nove de Julho University (UNINOVE), São Paulo, SP, Brazil.

The affiliation for the eighteenth author is incorrect. Mayana Zatz is not affiliated with #2 but with #3 Division of Human Genome Research Center, Biosciences Institute, University of São Paulo, São Paulo, SP, Brazil.

The affiliation for the last author is incorrect. Ana Paula Ligeiro de Oliveira is not affiliated with #3 but with #2 Laboratory of Pulmonary and Exercise Immunology–LABPEI, Nove de Julho University (UNINOVE), São Paulo, SP, Brazil.
