# Supplementary figures and images for: Human Tubal-Derived Mesenchymal Stromal Cells Associated with Low Level Laser Therapy Significantly Reduces Cigarette Smoke–Induced COPD in C57BL/6 mice
Source: PLoS One. 2015 Aug 31;10(8):e0136942. doi: 10.1371/journal.pone.0136942 (PMC4554986; doi:10.1371/journal.pone.0136942)

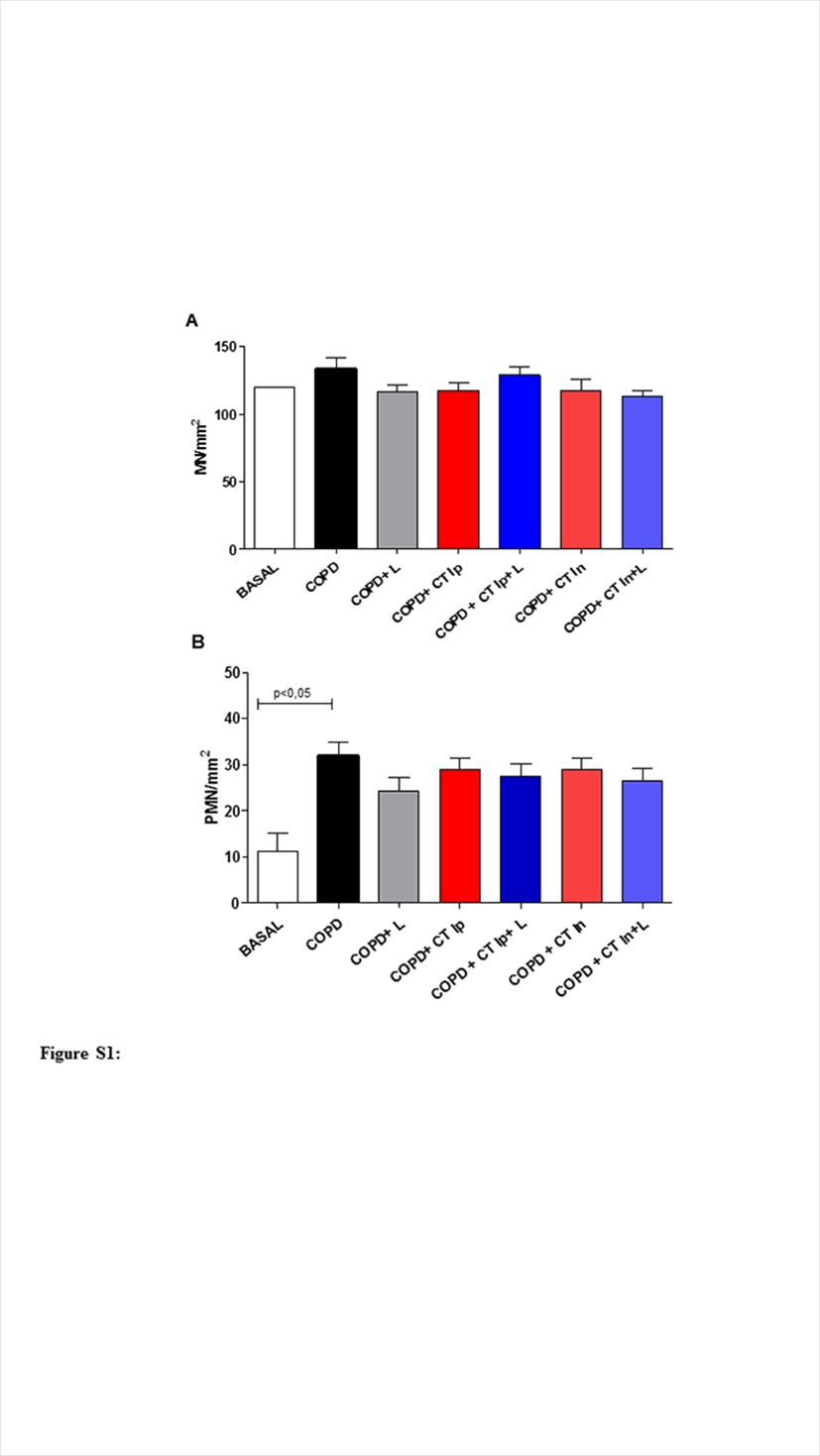

Supplement: S1 Fig — COPD animals were submitted to therapeutic protocols as described in materials and methods. Further, all animals were euthanized and lungs were obtained for histomorphometric analysis. In (A) mononuclear and (B) polymorphonuclear cells per mm2 of tissue. Data representative from two experiments. n = 5–8 animals per group. One-way ANOVA. (TIF) [file pone.0136942.s001.tif]

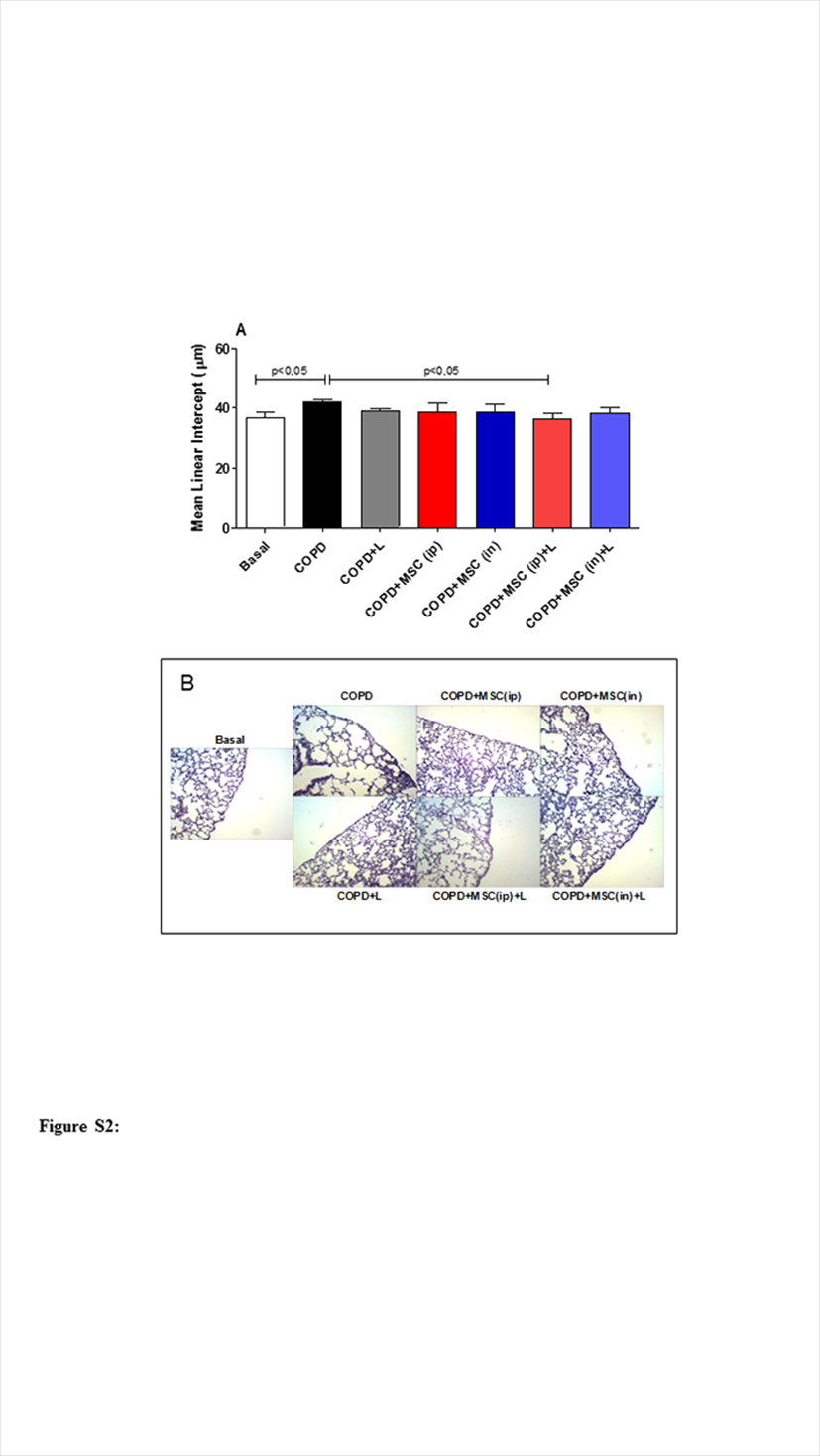

Supplement: S2 Fig — COPD animals were submitted to therapeutic protocols as described in materials and methods. Further, all animals were euthanized and lungs were obtained for mean linear intercept in central and peripheral measures of both lungs. In A) representative graphs and B) photomicrographs of haematoxylin- and eosin-stained pulmonary parenchyma. Data representative of two experiments. n = 5–8 animals per group. One-way ANOVA. (TIF) [file pone.0136942.s002.tif]

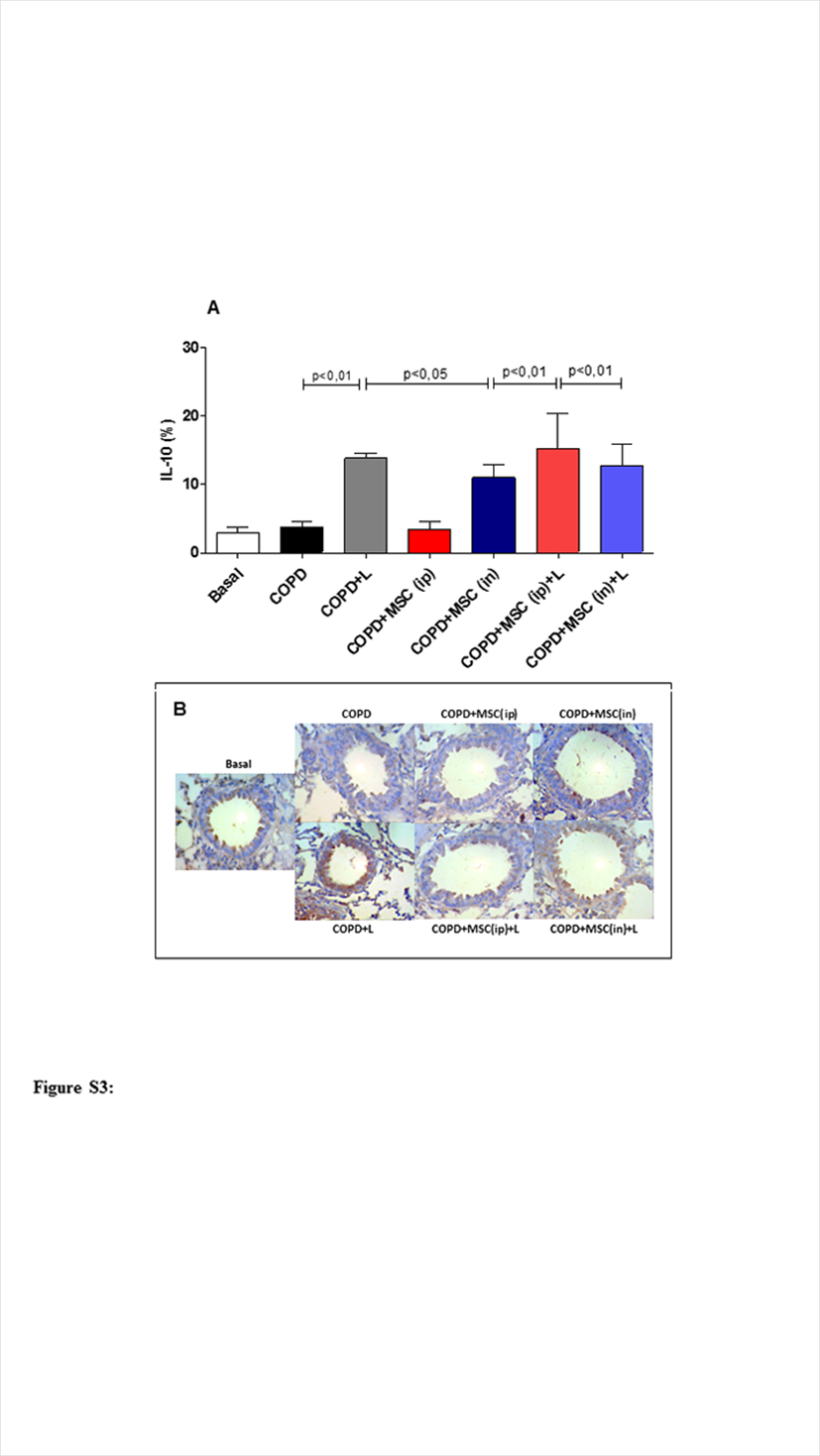

Supplement: S3 Fig — COPD animals were submitted to therapeutic protocols as described in materials and methods. Further, all animals were euthanized, lungs obtained and sections were stained with anti-IL-10. In A) representative graphs and B) photomicrographs of immunohistochemistry stained sections. Data representative of two experiments. n = 5–8 animals per group. One-way ANOVA. (TIF) [file pone.0136942.s003.tif]
